# Ferroptosis in hepatocellular carcinoma, from mechanism to effect

**DOI:** 10.3389/fonc.2024.1350011

**Published:** 2024-03-05

**Authors:** Shuang Jiang, Guangcong Zhang, Yanan Ma, Dongyu Wu, Da Xie, Songke Zhou, Xuemei Jiang

**Affiliations:** ^1^ Department of Gastroenterology, Hainan General Hospital (Affiliated Hainan Hospital of Hainan Medical University), Haikou, China; ^2^ Department of Gastroenterology and Hepatology, Zhongshan Hospital of Fudan University, Shanghai, China

**Keywords:** ferroptosis, hepatocellular carcinoma, lipid peroxidation, drugs, radiotherapy, immunotherapy

## Abstract

Hepatocellular carcinoma (HCC) is a prevalent malignant tumor worldwide, characterized by high malignancy and rapid progression. Most cases are diagnosed at intermediate to advanced stages. Current treatment methods have limited efficacy, resulting in high recurrence rates and poor prognosis. Radical hepatectomy remains the primary treatment for HCC, complemented by radiotherapy, chemotherapy, targeted therapy, and immunotherapy. Despite significant improvement in patient prognosis with radical hepatectomy, the five-year survival rate post-surgery remains low; thus necessitating exploration of more effective therapeutic approaches. Ferroptosis is a recently discovered form of cell death that can modulate the occurrence and development of HCC through various mechanisms. This article aims to elucidate the mechanism of ferroptosis and its impact on HCC development to provide novel insights for diagnosis and treatment.

## 1 Introduction

Hepatocellular carcinoma (HCC) is the most common pathological type of primary liver cancer. Based on the 2020 global Cancer statistics, HCC has a global incidence of 4.7% and a mortality rate of 8.3%. In China, the incidence and mortality rates are higher at 9.0% and 13% respectively (1). Currently, the diagnosis of HCC relies primarily on imaging techniques, including abdominal ultrasound (US), contrast-enhanced computed tomography (CT), and magnetic resonance imaging (MRI), as well as serological markers such as Alpha-fetoprotein (AFP), liquid biopsy (such as circulating tumor DNA gene sequencing and methylation), metabolomics, and glycomicomics (2). The most commonly used methods for detecting HCC are US and serological content analysis of AFP. However, their accuracy is limited, US has an overall sensitivity of 84% in clinical screening and diagnosis of HCC patients, but its sensitivity is affected by the tumor diameter (3); the sensitivity of serum AFP in the diagnosis of early HCC ranges from 45.3% to 62.0%, with a specificity of 87% to 93% (4). Furthermore, due to the insidious nature and lack of specific symptoms in the early stages of HCC, it is difficult to screen using routine examinations. The clinical diagnosis of HCC is mostly made at an advanced stage. Therefore, it is essential to explore more effective treatments for liver cancer to reduce the medical and economic burden on society. In 2012, a mode of cell death that is dependent on iron was officially named ‘ferroptosis’. Studies have shown that ferroptosis plays a significant role in HCC (5). This article aims to discuss the regulatory mechanism of ferroptosis and its application in the treatment of HCC, providing new ideas for improving the level of HCC treatment.

## 2 Characteristics of ferroptosis

The morphological manifestations of ferroptosis are as follows: the mitochondrial volume of cells in which ferroptosis occurs shrinks and the cells become smaller. The mitochondrial crista decrease or disappear, the density of the membrane increases, and the inner membrane aggregates, resulting in rupture of the mitochondrial outer membrane. Biochemically, the aggregation of iron ions, the accumulation of reactive oxygen species (ROS) and the excessive accumulation of lipid peroxides are observed (6). When iron metabolism is disordered, it can mediate lipid peroxidation and eventually generate lipid peroxides, resulting in the accumulation of lipid peroxides, damage to the body’s cell membrane system, stability of the lipid bilayer is destroyed, the cell membrane is ruptured, and finally cells die due to ferroptosis (7).

## 3 Regulation of ferroptosis and its role in HCC

**Figure 1:**
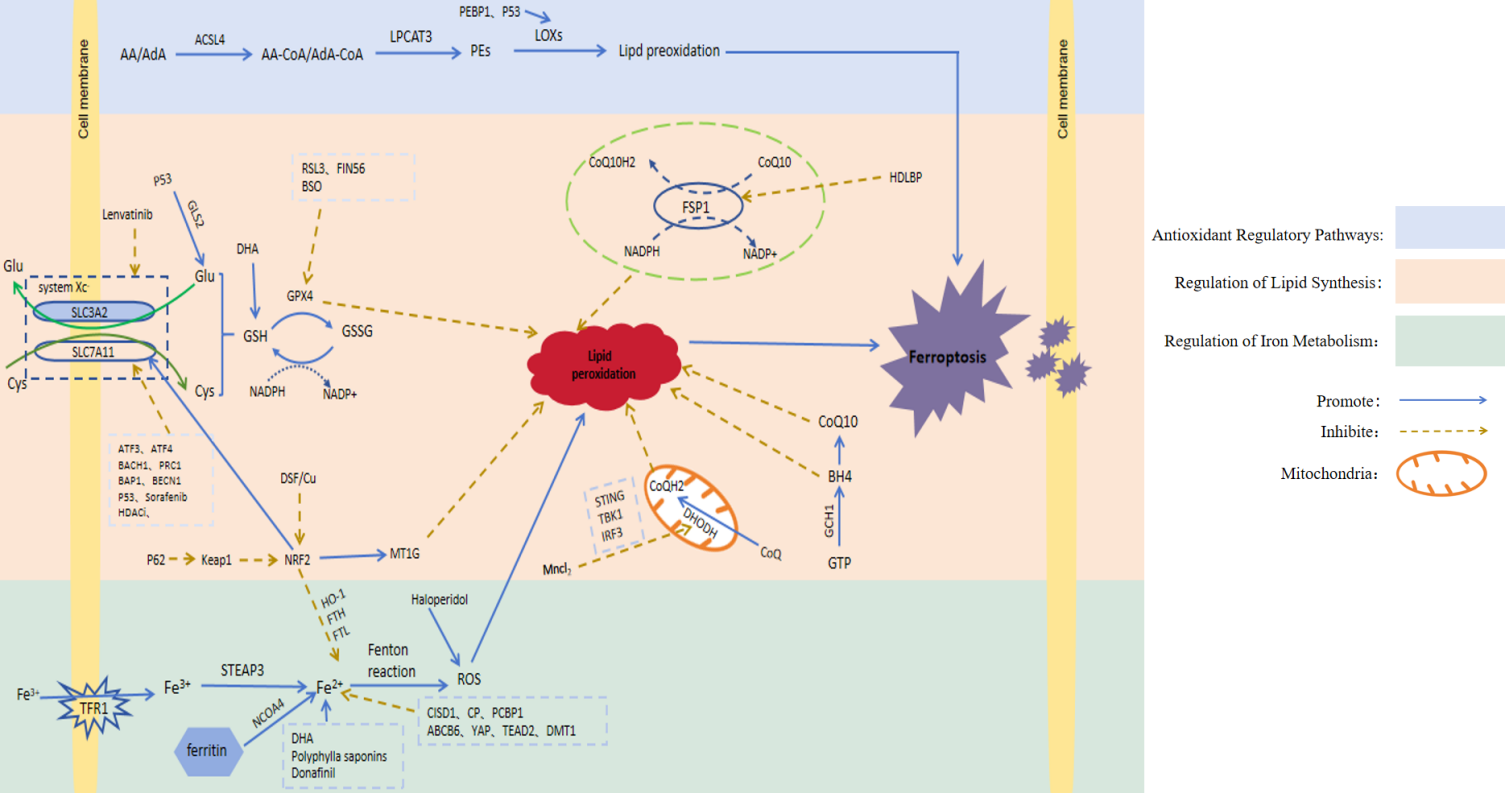
Signaling pathways involved in ferroptosis regulation. AA, Arachidonic acid; AdA, Adrenoic acid; LPCAT3, Lysophosphatidylcholine acyltransferase 3; PEs, Containing phosphatidylethanolamine from AA or AdA; Glu, Glutamate; Cys, Cysteine; System Xc-, Cystine/glutamate countertransporter system Xc; SLC7A11, Solute carrier family 7 member 11; SLC3A2, Solute carrier family 3 member 2; GLS2, Glutaminase 2; GSH, Glutathione; GPX4, Glutathione peroxidase 4; GSSG, Oxidised glutathione; NAD(P)H, Nicotinamide adenine dinucleotide (phosphate); RSL3, GPX4 inhibitor; FIN56, Ferroptosis inducers; ATF3, Activatingtranscriptionfactor 3; ATF4, Activatingtranscriptionfactor 4; BACH1, Basic leucine zipper transcription factor 1; PRC1, Protein regulator of cytokinesis 1; BECN1, Recombinant Human Beclin 1; BAP1, BRCA1-associated protein 1; Keap1, Kelch-like ECH-associated protein 1; NRF2, Nuclear factor erythroid-derived 2; MT-1G, Metallothionein; CoQ10H2, Ubiquinol; CoQ10, Coenzyme Q10; FSP1, Ferroptosis suppressor protein 1; HDLBP, High-density lipoprotein binding protein; IFN, Type-I interferon; DHODH, Dihydroorotate dehydrogenase; GTP, Guanosine triphosphate; GCH1, Guanosine triphosphate cyclohydrolase 1; BH4, Tetrahydrobiopterin; TFR1, Transferrin receptor 1; STEAP3, Six-transmembrane epithelial antigen of the prostate3; NCOA4, Nuclear receptor coactivator 4; FTH, Ferritin heavy chain; FTL, Ferritin light chain; HO-1, Heme oxygenase-1; CISD1, CDGSH iron-sulfur domain 1; CP, Ceruloplasmin; PCBP1, Poly C binding protein 1; ABCB6, ATP-binding cassette sub-family B member 6; YAP, Yes-associated protein; TEAD2, Transcriptional enhanced associate domain 2; DMT1, Divalent metal transporter1; ROS, Reactive oxygen species; DHA, Dihydroartemisinin; BSO, Auranofin and buthionine sulfoxide; HDACi, Histone deacetylase inhibitor; DSF/Cu, Disulfiram/copper.

It has been found that ferroptosis plays an important regulatory role in HCC. Here, we will take three regulatory pathways of ferroptosis as the starting point, and briefly describe the action mechanisms of these three pathways, namely the relevant regulators (**Figure 1**; **Table 1**).

**Table 1:** Molecules involved in the ferroptosis regulatory pathway.

	Molecule	Mechanism
Regulators of the iron metabolism pathway	CISD1 (8)	Inhibition of iron uptake
	CP (9)	Influence of Fe content
	PCBP1 (10)	Influence of Fe content
	ABCB6 (11)	Reduce the Fe^2+^ content
	YAP (12)	The expression of TFRC was increased
	TEAD2 (13)	Inhibition of Fe accumulation
	NCOA4 (14)	Degrades ferritin and increases the Fe^2+^ content
	DMT1 (15)	Inhibition of Fe uptake
Modulators of lipid metabolic pathways	ACSL4 (16)	The initiation of PUFA to undergo peroxidation
	PEBP1 (17)	Promoting the production of LOXs
Modulators of the antioxidant pathway	RSL3 (18)	Inhibition of GPX4 activity
	FIN56 (18)	Inhibition of GPX4 activity
	ATF3 (19)	Inhibition of SLC7A11 expression
	ATF4 (20)	Inhibition of SLC7A11 expression
	BACH1 (21)	Inhibition of SLC7A11 expression
	PRC1 (22)	Inhibition of SLC7A11 expression
	BAP1 (22)	Inhibition of SLC7A11 expression
	BECN1 (23)	Inhibition of SLC7A11 expression
	MT-1G (24)	Accelerate the consumption of GSH
	HDLBP (25)	Competitive elimination of TRIM69-dependent FSP1 polyubiquitination degradation
	MnCl_2_ (26)	Inhibition of DHODH expression

CISD1, CDGSH iron-sulfur domain 1; CP, Ceruloplasmin; PCBP1, Poly C binding protein 1; ABCB6, ATP-binding cassette sub-family B member 6; YAP, Yes-associated protein; TEAD2, Transcriptional enhanced associate domain 2; NCOA4, Nuclear receptor coactivator 4; DVMT1, Divalent metal transporter1; ACSL4, Acyl-coa synthetase long-chain familymember4; PEBP1, Phosphatidylethanolamine binding protein 1; RSL3, GPX4 inhibitor; FIN56, Ferroptosis inducers; ATF3, Activatingtranscriptionfactor 3; ATF4, Activatingtranscriptionfactor 4; BACH1, Basic leucine zipper transcription factor 1; PRC1, protein regulator of cytokinesis 1; BAP1, BRCA1-associated protein 1; BECN1, Recombinant Human Beclin 1; MT-1G, Metallothionein; HDLBP, High-density lipoprotein binding protein.

### 3.1 Regulation of iron metabolism

Iron is an essential trace element in cells. It participates in the body’s metabolic processes in many ways and plays a crucial role in development of the body. Studies have shown that cancer cells increase intracellular iron concentration to promote cells through a variety of mechanisms (27). Ferrum is a redox-active metal due to its ability to easily gain or lose electrons. In the body, ferrum exists in two forms: A. Fe^3+^ is bound to ferritin and is responsible for participating in transport in the body. B. Fe^2+^ is absorbed by the body [5]. Specifically, Fe^3+^ binds to transferrin receptor 1 (TFR1) located on the cell membrane in order to enter the cell. In the cell, Fe^3+^ is reduced by iron oxidoreductase (six-transmembrane epithelial antigen of the prostate 3, STEAP3) to Fe^2 +^ with higher catalytic activity, which is extremely unstable and can transfer electrons to intracellular oxygen. Peroxidation of lipids to lipid peroxides; On the other hand, in a weak acidic environment, Fe^2+^ can also generate lipid peroxides through the Fenton reaction by activating hydrogen peroxide (H_2_O_2_) in the body and generating hydroxyl radicals. Hydroxyl radicals are the most active free radicals of ROS, which can peroxidize lipids and generate lipid peroxides. Excessive lipid peroxides destroy the integrity of the cell membrane and eventually induce ferroptosis (28). As the most important organ for iron storage and iron metabolism in the body, the liver can regulate the iron content and the occurrence of ferroptosis in HCC cells, which may be a new treatment strategy for HCC patients. For example, CDGSH iron-sulfur domain 1 (CISD1) is an iron-containing mitochondrial outer membrane iron-sulfur protein. Studies have shown that this protein can further inhibit the occurrence of lipid peroxidation and reduce the accumulation of lipid peroxides by inhibiting mitochondrial iron uptake in HCC cells (8). Ceruloplasmin (CP) is a Fe^2+^ chelating glycoprotein. The depletion of CP can lead to Fe^2+^ accumulation and promote ferroptosis induced by erastin and RSL3 (9). Poly C binding protein 1 (PCBP1) is a multifunctional protein. One of its functions is to act as an iron chaperone for cellular solutes, binding iron and transferring it to receptor proteins in mammalian cells, thereby affecting iron content. PCBP1 deficiency mediates ferroptosis in HCC cells by increasing iron accumulation and ROS production (10). ABCB6, a member of the ATP-binding cassette (ABC) transporters, is one of the largest families of membrane proteins in most organisms. It has been shown that ABCB6 is highly expressed in HCC cells, and iron-containing porphyrin can be transported to the outside of cells under the action of ABCB6, thereby reducing intracellular iron content. At this time, ferroptosis in HCC cells was inhibited (11). The increased activity of Yes-associated protein (YAP) induces the expression of transferrin receptor (TFRC), and the overexpression of TFRC promotes iron absorption and increases iron content in HCC cells, thereby enhancing the sensitivity of HCC to ferroptosis (12). TEAD2 is a member of the transcriptional enhanced associate domain (TEAD) family (13). TEAD2 is significantly up-regulated in HCC, which plays an inhibitory role in the ferroptosis of HCC cells mainly by inhibiting the accumulation of iron. Ferritin can bind to nuclear receptor coactivator 4 (NCOA4) of autophagy and be transported to lysosomes, where ferritin is degraded and a large amount of Fe^2+^ is released, a process called ferroautophagy (14). Therefore, increasing the degradation of ferritin can increase the content of free iron, thereby promoting ferroptosis of cells. Divalent metal transporter1 (DMT1) shuttles iron across the plasma membrane, and most cells uptake iron through endocytosis; inhibition of DMT1 results in the production of ROS, lipid peroxidation, and cell death (15). Regulation of the iron metabolism pathway is the initiating factor of ferroptosis, and the accumulation of iron is one of the key factors in the ferroptosis of HCC cells. The increase in iron intake and the decrease in iron consumption or loss in the body due to various reasons can cause excessive intracellular iron accumulation, thereby promoting ferroptosis.

### 3.2 Regulation of lipid synthesis

The formation of lipid peroxides by lipid peroxidation is a key process in the induction of ferroptosis. Thus, lipid peroxides are the most dominant feature of ferroptosis. *In vivo*, the substrates of cell peroxidation are mainly lipids containing polyunsaturated fatty acids (PUFA). Lipid substances containing acyl groups of unsaturated fatty acids can be formed into lipid peroxides by enzymatic or non-enzymatic reactions. The first enzymatic reaction pathway: Arachidonic acid (AA) and adrenoic acid (AdA) are the most common polyunsaturated fatty acids in the body. These two compounds can first be catalyzed by acyl-CoA synthetase long-chain family member4 (ACSL4) to produce acyl-CoA derivatives. Then lysophosphatidylcholine acyltransferase 3 (LPCAT3) catalyzes the formation of phosphatidylethanolamine (PE). Finally, it is oxidized to toxic lipid peroxides by lipoxygenases (LOXs). Second, in a weak acid environment, PUFA is catalyzed by Fe^2+^ through the Fenton reaction to generate ROS, which then promote lipid peroxidation to generate lipid peroxides (16). In the body, the accumulation of lipid peroxides exceeds the body’s tolerance, which promotes the occurrence of ferroptosis. Other studies have reported that monounsaturated fatty acids (MUFA), unlike PUFA, have been shown to negatively regulate ferroptosis (29). However, the mechanism by which exogenous MUFA inhibit ferroptosis has not been fully elucidated. Liver is an important organ for lipid synthesis and metabolism. Ferroptosis of HCC cells is closely related to abnormal lipid metabolism in the liver. Previous studies (30) have shown that in HCC cells, ACSL4 can be activated by protein kinase Cβ2. Activated ACSL4 can initiate PUFA peroxidation, thereby triggering a series of lipid peroxidation reactions, resulting in increased production of lipid peroxides and the induction of ferroptosis in HCC cells. The above research provides us with inspiration: First, we can increase the content of PUFA such as AA and adrenal acid in the body. For example, AA is easily absorbed from meat intake, but AA can also be converted from other compounds. For example, the PUFA from plant food is mainly PUFA phospholipid containing linoleic acid (LA). LA can be mediated by fatty acid desaturase 1 and 2 (FADS1, FADS2) and elongase of very long chain fatty acids 5 (ELOVL5). After a series of enzymatic reactions, AA is finally generated (31, 32). Second, it can promote lipid peroxidation and increase the generation of lipid peroxides by increasing the content of key enzymes of peroxidation or catalyzing the reactions in which key enzymes are involved, such as phosphatidylethanolamine binding protein 1 (PEBP1). Ying et al. found that PEBP1 expression was low in liver cancer, indicating that this protein was negatively correlated with the degree of malignancy in human liver cancer, further reflecting the important role of PEBP1 in the occurrence and development of HCC (17).

### 3.3 Antioxidant regulatory pathways

The occurrence of ferroptosis is inseparable from the peroxidation reaction, which is closely related to the antioxidant regulatory pathway. Several antioxidant pathways have been found to be involved in ferroptosis.

#### 3.3.1 GSH and GPX4 pathways

The glutathione (GSH)/glutathione peroxidase 4 (GPX4) pathway is the first identified molecular pathway that can regulate ferroptosis through antioxidants, which is also known as the classical pathway of ferroptosis. GSH is a small molecule with antioxidant activity in the body, and is composed of glutamate, glycine and cysteine. GSH protects cells from oxidative damage through antioxidant action. GSH is converted to glutathione oxidized (GSSG) under the catalytic action of GPX4, which reduces ROS in the body and reduces cell damage caused by excessive accumulation of ROS. From this point of view, GPX4 is used as a reducing agent in the anti-peroxidation reaction, and GSH has an antioxidant effect on GPX4, the antioxidant effect of the two complement each other and depend on each other. In addition, some studies have found that GPX4 can directly reduce lipid peroxide to the corresponding alcohol or reduce H_2_O_2_ to water. It is well known that H_2_O_2_ is a strong oxidant, if H_2_O_2_ is depleted, the peroxidation reaction in the body will be weakened, the formation of lipid peroxidation will be reduced, and the occurrence of ferroptosis will be inhibited (18). Therefore, the activities of GPX4 and GSH are inhibited by various pathways, the ability of anti-oxidation in cells is weakened, oxidation is dominant, and lipid is peroxidized, thus inducing ferroptosis of cells (33). GPX4 is the only enzyme found to protect mammalian biofilms from peroxidation-induced cell membrane damage because it directly reduces lipid peroxides to the related product, glutathione peroxidase (34). Li et al. (35) showed that compounds such as DSF/Cu can directly inhibit GPX4 activity and induce ferroptosis.

System Xc- is a type of amino acid reverse transporter attached to the body’s cell membrane, and its main function is the reverse transport of cystine and glutamate. The main components of System Xc- are SLC7A11 and SLC3A2. Of these, SLC7A11 is mainly responsible for transporting cystine and glutamate and is associated with System XC-transport activity, while SLC3A2 mainly functions as a chaperone protein. Glutamate is mainly found in the cell, whereas cystine is mainly found outside the cell. System Xc- can expel one molecule of glutamate from the cell, while also transporting one molecule of cystine from the outside to the cell. The reversed cystine is rapidly reduced to oxidation-active cysteine, which reacts with glutamate and glycine as substrates to produce GSH (36). Thus, when the amount of System Xc- in the cell is insufficient, it leads to insufficient GSH production, resulting in a consequent decrease in GPX4 oxidative activity. This provided us with new insight and suggested that System Xc- overexpression in human hepatocytes could significantly inhibit ferroptosis in HCC cells. Decreased GSH synthesis and inhibition of GPX4 activity for various reasons can promote ferroptosis in HCC cells. For example, ATF3, ATF4, BACH1, PRC1, BAP1, and BECN1 can inhibit the expression of SLC7A11 (19–23); Knockdown of metallothionein (MT-1G) accelerated GSH consumption (24). The above factors and compounds can weaken the antioxidant capacity *in vivo* through the GSH/GPX4 pathway, thereby improving the sensitivity of HCC cells to ferroptosis.

#### 3.3.2 The NAD(P)H/FSP1/CoQ10 pathway

This pathway is another antioxidant pathway independent of the classical ferroptosis pathway. Ferroptosis suppressor protein 1 (FSP1) is an oxidoreductase of Coenzyme Q10 (CoQ10), which is an important factor in the regulatory mechanism of oxidative phosphorylation *in vivo*. It exerts antioxidant effects mainly by reducing NAD(P)H-dependent ubiquinone (CoQ) to panthenol with antioxidant activity (37), In the case of GPX4 deficiency, FSP1 can protect cells from ferroptosis by regenerating CoQ10 using NADH. On the other hand, FSP1-deficient cancer cells were found to be sensitive to ferroptosis activator-induced cell death, and intracellular administration of CoQ10 failed to reverse FSP1-deficient mediated ferroptosis. In contrast, FSP1 activates the transporter required endosomal sorting complex (ESCRT)-III-dependent membrane repair pathway. This mechanism is driven by impaired FSP1-dependent membrane repair machinery, but not by FSP1-dependent panthenol production. This suggests that FSP1 directly inhibits ferroptosis independent of panthenol production in HCC (38). Therefore, ferroptosis of HCC cells can be regulated by modulating the expression of the NAD(P)H/FSP1/CoQ10 pathway. For example, high-density lipoprotein binding protein (HDLBP) is significantly increased in HCC, which can bind and stabilize the long non-coding (lnc)RNA lncFAL (ferroptosis-related lncRNA). RNA lncFAL can directly bind to FSP1 and competitively eliminate TRIM69-dependent FSP1 polyubiquitination degradation to increase ferroptosis susceptibility in HCC (25).

#### 3.3.3 The P62-Keap1-NRF2 pathway

The P62-kelch-like ECH-associated protein 1 (Keap1)-transcription factor nuclear factor erythroid-derived 2 (NRF2) signaling pathway protects the body from oxidative stress by inducing cells to produce protective factors. However, NRF2 plays a major role as an antioxidant. NRF2 is normally tightly bound to Keap1, and NRF2 is maintained at a low dissociation level *in vivo*. When the body is in a state of oxidative stress, NRF2 will immediately dissociate from Keap1. At the same time, NRF2 will be rapidly transported to the nucleus to activate the expression of target genes with antioxidant activity in the promoter region of the nucleus, and resist the oxidative stress by transcription to avoid cell damage caused by ferroptosis induced by cells under strong oxidative stress for a long time (39). P62 is an autophagy adaptor protein, which can promote the activation of NRF2 and the antioxidant effect by directly inhibiting Keap1. In addition, NRF2 can also promote the expression of GPX4, and the target mechanism of action is to regulate the expression of SystemXc-. NRF2, also induces the expression of MT-1G through the cystathionine pathway and inhibits ferroptosis. Therefore, it has been shown that inhibition of MT-1G expression can enhance the anti-cancer activity of sorafenib in the treatment of HCC (40). This provides a promising strategy for improving sorafenib resistance. NRF2 activation can further mediate HCC cells against ferroptosis by regulating iron-related genes such as HO-1, FTH, and FTL, preventing iron accumulation and further inhibiting the production of ROS (41).

#### 3.3.4 Inhibition of DHODH pathway induction

Dihydroorotate dehydrogenase (DHODH) is a newly discovered antioxidant pathway that can regulate ferroptosis, but it mainly plays a role in regulating ferroptosis in mitochondria. DHODH, an enzyme widely expressed in the inner mitochondrial membrane, is the fourth catalytic rate-limiting enzyme for *de novo* pyrimidine synthesis and is involved in the regulation of *de novo* pyrimidine nucleotide synthesis. DHODH inhibits ferroptosis in the inner mitochondrial membrane by oxidizing dihydroorotic acid to orotic acid and reducing ubiquinone (CoQ) to CoQH2 with antioxidant activity (42). Therefore, the addition of a DHODH inhibitor such as breqinar increases sensitivity to ferroptosis in the inner mitochondrial membrane, and the inactivation of DHODH induces widespread ferroptosis in cancer cells with low GPX4 expression (42). As shown in recent studies, MnCl_2_ can promote the expression of type I interferon by enhancing the phosphorylation levels of STING, TBK1 and IRF3, and then inhibit the expression of DHODH in tumor cells, leading to an increase in mitochondrial ROS and lipid peroxidation, thereby increasing the sensitivity of cells to ferroptosis (26). Li et al. found that in HCC, inhibition of DHODH expression could enhance the sensitivity of HCC cells to sorafenib-induced ferroptosis (43). This study provides a new idea for the future treatment of HCC by targeting ferroptosis.

#### 3.3.5 Blockade of GCH1/BH4 pathway induced ferroptosis

GTP Cyclohydrolase 1 (GCH1) is the first rate-limiting enzyme for *de novo* synthesis of tetrahydrobipterin (BH4), an antioxidant that protects cells from lipid peroxidation. On the other hand, BH4 is also a key enzyme in the synthesis of CoQ precursors, which plays a dual role in antioxidant activity (44). Epigenetic silencing of GCH1 promotes HCC cell growth by inhibiting the *de novo* synthesis of BH4 and activating the superoxide anion mediated ASK1/p38 signaling pathway, suggesting that targeting the GCH1/BH4 pathway may be a promising therapeutic strategy against HCC (45). Furthermore, overexpression of GCH1 in mouse fibroblasts significantly inhibited GPX4 induced ferroptosis. These results indicated that GCH1 overexpression could selectively inhibit the peroxidation of some unsaturated fatty acids and thus down-regulate ferroptosis (46). Therefore, blocking the pathway and inhibiting the expression of CPX4 can play a synergistic role in inducing ferroptosis, which could provide us with a new method for the future treatment of HCC: CPX4 inhibitors can be combined with GCH1/BH4 pathway inhibitors, and both play a role in inducing ferroptosis in HCC cells to fight tumor proliferation.

### 3.4 Other molecular pathways: the p53 signaling pathway

P53 is a tumor suppressor gene. It has been found that p53 can regulate ferroptosis of HCC cells through multiple pathways as follows (1): The expression of SLC7A11 is inhibited by transcription, the uptake of cystine is inhibited, the substrate of GSH is reduced, synthesis is insufficient, and the content of GSH is decreased, thus the activity of GPX4 in cells is inhibited, the antioxidant capacity *in vivo* is weakened, oxidation is dominant, and ferroptosis of HCC cells is promoted (47). (2) Regulation of induced spermidine/spermine N1-acetyltransferase (SAT1) gene expression, thereby promoting arachidonate-15-lipoxygenase (ALOX15), an enzyme that promotes lipid peroxidation, leading to ferroptosis (48). (3) Overexpression of Glutaminase 2 (GLS2), which is a key enzyme in the conversion of glutamine to glutamate, one of the three synthesis substrates of GSH, can inhibit the ferroptosis of HCC cells by increasing GSH synthesis (49). (4) Iron metabolism is regulated by ferredoxin reductase (FDXR). FDXR regulates iron metabolism through the transfer of electrons to cytochrome P450 by ferredoxin in mitochondria, and p53 can induce the transcriptional expression of FDXR. At the same time, FDXR promoted the translation of p53mRNA through intracellular iron regulatory protein 2, thereby increasing the level of p53 protein. Thus, FDXR and p53 are mutually regulated and can promote each other, and this FDXR-p53 loop has been shown to regulate ferroptosis in HCC cells by maintaining mitochondrial iron homeostasis (50). As mentioned above, p53 can regulate HCC cell development through a variety of mechanisms. New guidelines for the diagnosis and treatment of HCC can be provided using these multiple regulatory ideas.

## 4 Treatment of HCC based on ferroptosis

The induction of ferroptosis in HCC cells provides a new therapeutic option for HCC treatment. In the current treatment of HCC, some drugs have been found to exert anti-tumor effects by inducing cell ferroptosis (**Table 2**).

**Table 2:** Drugs that have been found to regulate ferroptosis in HCC.

Drug	Mechanism	Clinical stages
Sorafenib (37, 51–54)	Suppress System Xc-	Advanced
DHA (55–59)	increased Fe content and inhibited GSH synthesis	Advanced
Polyphylla saponins (60)	Increase Fe content	/
Haloperidol (61)	promotes the accumulation of ROS	/
BSO (62)	GPX4 generation is inhibited	/
Lenvatinib (63)	Suppress System Xc-	Advanced
Donafinil (64, 65)	increase intracellular Fe^2+^ levels	Advanced
HDACi (66, 67)	Suppress SLC7A11	Advanced
DSF/Cu (68)	Inhibit NRF2 activity	/

DHA, Dihydroartemisinin; BSO, Auranofin and buthionine sulfoxide; HDACi, Histone deacetylase inhibitor; DSF/Cu, Disulfiram/copper.

"/" indicates that the drug is relatively early and has not been tested in clinical trials.

### 4.1 Sorafenib

Sorafenib is the first targeted drug approved for clinical application in the treatment of patients with advanced liver cancer worldwide. Previously, as a multikinase inhibitor, sorafenib was considered to play an anti-cancer effect mainly through an enzymatic reaction. However, in recent years, the anti-cancer effect of sorafenib has been confirmed to be mainly due to the induction of ferroptosis in HCC cells. There are two known ways sorafenib induces ferroptosis: (1) silencing the retinoblastoma (Rb) gene in HCC cells by transfection of short hairpin RNA. Silencing of Rb protein can increase the sensitivity of HCC cells to ferroptosis, thereby enhancing its anti-cancer effect (51). (2) By inhibiting the production of System Xc-, the antioxidant capacity of HCC cells is weakened, oxidation is dominant, the production of lipid peroxides is increased, and HCC cells are more susceptible to ferroptosis (51). At the same time, resistance mechanisms related to sorafenib are also involved in the regulation of ferroptosis: Some studies have found that following sorafenib treatment, p62 protein can inactivate Keap1 and inhibit the degradation of NRF2, resulting in an increase of NRF2 content and the inhibition of iron accumulation by activating iron-related genes, thereby reducing the generation of ROS and inhibiting the occurrence of ferroptosis. In addition, NRF2 activation increased MT-1G expression in HCC cells and inhibited lipid peroxidation by blocking GSH consumption. This suggests that inhibition of NRF2 expression may enhance the efficacy of sorafenib in the treatment of HCC. For example: A. Quiescin Q6 Sulfhydryl Oxidase 1 (QSOX1) is a disulfide catalyst that inhibits epidermal growth factor (EGF)-induced EGF receptor (EGFR) activation by promoting ubiquitin-mediated EGFR degradation and accelerating its intracellular endosomal trafficking, thereby inhibiting NRF2 activity. Furthermore, it promotes sorafenib-induced ferroptosis in HCC cells (52). B. Gao et al. found that the YAP/TAZ and ATF4 genes in the nucleus were highly expressed in HCC cells with sorafenib resistance, and the expression of SLC7A11 in these cells was increased, which reduced the expression level of ROS and the production of lipid peroxides, and inhibited the ferroptosis of HCC cells (53). C. Inhibition of phosphoseryl-tRNA kinase (PSTK) increased the sensitivity of HCC cells to chemotherapy. PSTK has been implicated in the inhibition of chemotherapy-induced ferroptosis in HCC cells, and depletion of PSTK leads to GPX4 inactivation and disruption of GSH metabolism due to inhibition of selenocysteine and cysteine synthesis, thereby enhancing ferroptosis induction after targeted therapy. Punicalin, a drug used to treat hepatitis B virus, was identified as a possible PSTK inhibitor of peroxide accumulation and negatively regulated ferroptosis in HCC cells. Inhibition of MT-1G expression enhanced the anti-cancer activity of sorafenib by inducing ferroptosis *in vitro* and *in vivo*. This finding indicates that MT-1G is a novel regulator of ferroptosis in HCC and demonstrates a novel molecular mechanism of sorafenib resistance (37). It can be seen that NRF2 status has an important role in influencing the therapeutic efficacy of ferroptosis in HCC treatment, showing synergistic efficacy when applied together with sorafenib for HCC treatment *in vitro* and *in vivo* (54).

### 4.2 Dihydroartemisinin

Arteannuin (ART) is a malaria drug discovered by the Chinese scientist Tu Youyou (55, 56). Dihydroartemisinin (DHA) is one of the many derivatives of ART. Studies have shown that DHA can not only be used for the treatment of malaria, but also has anti-tumor effects. ART is considered to be one of the many inducers of ferroptosis, which can induce ferroptosis in a variety of malignant tumor cells and plays an anti-tumor role. It has been found that DHA induces ferroptosis in cancer cells mainly through the following mechanisms: (1) by increasing the degradation of ferritin by transferrin, lysosomes and autophagosomes, and directly increasing the concentration of Fe in the labile iron pool, thereby promoting the accumulation of ROS and inducing ferroptosis (57). (2) By inhibiting the synthesis of GSH and thus the activity of GPX4 is then inhibited, the antioxidant effect is lost, and oxidation is enhanced, which is conducive to the occurrence of ferroptosis. (3) Activation of the ER stress response, PERK-ATF4-HSPA5 pathway and ATF4-CHOP-CHAC1 pathway affect Fe content to regulate ferroptosis. (4) By affecting the electron transport chain in mitochondria, it promotes the generation of ROS and increases the sensitivity of cells to ferroptosis (58). (5) Mammalian 15-lipoxygenase/phosphoethanolamine binding protein-1 (15-LO) oxidizes free PUFA with the help of PEBP1 to form the PEBP1/15-LO complex. DHA inhibits the ubiquitin-proteasome pathway of PEBP1 to increase its protein level and bind to 15-LO. Furthermore, DHA promoted ferroptosis induced by lipid peroxidation of HCC cell membranes, thereby exerting the anti-HCC activity of DHA (17). In conclusion, DHA induces ferroptosis through multiple pathways, which fully illustrates the potential role of artemisinin in the treatment of tumors. At present, DHA has been approved for clinical trials in cancer, and clinical trials of artemisinin in the treatment of HCC are under investigation (59). It is believed that artemisinin and its derivatives will be used in the treatment of clinical tumors in the near future.

### 4.3 Others

(1) Polyphylla saponins, are Chinese herbal medicine components (60). Polyphyllin can increase the iron content of HCC cells by increasing ferritin phagocytosis, thereby inducing ferroptosis of HCC cells. (2) Haloperidol is an antipsychotic drug, but its combination can promote sorafenib-induced ferroptosis in HCC cells. As a sigma receptor 1 antagonist, haloperidol mainly promotes the accumulation of intracellular ROS, thereby promoting lipid peroxidation and inducing ferroptosis. Haloperidol combined with sorafenib has been shown to enhance sorafenib-induced ferroptosis in the treatment of HCC patients (61). (3) The combination of auranofin and buthionine sulfoxide (BSO) or the treatment combination of sub-toxic concentrations of erastin and BSO, both inhibited GPX4 production and affected HCC cell ferroptosis (62). (4) Lenvatinib is another targeted drug that can be used for the treatment of advanced HCC. In 2018, lenvatinib was officially approved by the US Food and Drug Administration for the first-line treatment of patients with advanced HCC. The mechanism of ferroptosis induction mainly involves inhibiting fibroblast growth factor receptor-4 (FGFR4) to inhibit System Xc- expression and increase ROS accumulation, thereby inducing ferroptosis in HCC cells (63). (5) Donafinil, a deuterated derivative of sorafenib, is an approved standard treatment for patients with advanced HCC. Preclinical studies using small-molecule inhibitors and drug-induced CRISPR libraries have shown that donafinil acts synergistically with GSK-J4 to promote HMOX1 expression and increase intracellular Fe^2+^ levels, ultimately leading to ferroptosis in HCC (64, 65). (6) Histone deacetylase inhibitors (HDACi) can inhibit SLC7A11, induce ROS accumulation and induce ferroptosis in tumor cells (66). Of these, sodium butyrate (NaB) has been shown to inhibit the proliferation of HCC cells as a class I HDACi (67). (7) DSF/Cu can disrupt mitochondrial homeostasis, increase the free iron pool, enhance lipid peroxidation, and eventually lead to iron-induced cell death. Mechanistically, DSF/Cu can enhance the cytotoxicity of sorafenib and inhibit tumor growth *in vitro* and *in vivo* by inhibiting NRF2 (68).

### 4.4 Radiotherapy

Radiotherapy is considered beneficial for patients with unresectable or advanced HCC. It is an important part of the treatment of these patients. Studies have demonstrated that radiotherapy can trigger ferroptosis in cancer cells (69). Radiotherapy induces ROS production and ACSL4 expression, leading to lipid peroxidation and ferroptosis. As an adaptation, radiotherapy also induces overexpression of death suppressor genes such as SLC7A11 and GPX4. Tumor cells overexpressing SLC7A11 and GPX4 show marked resistance to radiotherapy. Sensitization of radioresistant cancer cells to radiotherapy can be achieved through inactivation of SLC7A11 or GPX4 with ferroptosis inducers (70). For instance, suppressor of cytokine signaling 2 (SOCS2) is a potential target for radiosensitization in HCC (71), the sensitivity of HCC cells to radiotherapy can be increased by overexpressing SOCS2, which promotes the degradation of SLC7A11. The protein complex regulated by nuclear respiratory factor 1/Ras-associated nuclear protein/dihydrolipoamide dehydrogenase can be targeted for radiation to enhance the radiosensitivity of HCC cells by inducing ferroptosis (72). Ionizing radiation reduces the expression of Copper metabolism MURR1 domain 10 (COMMD10), inhibits the ubiquitin degradation of HIF1α, and impairs its binding to HIF1α. While inhibiting ferroptosis in HCC, it promotes nuclear translocation of HIF1α and transcription of CP and SLC7A11. Additionally, elevated CP promotes HIF1α expression by reducing iron, forming a positive feedback loop. In conclusion, these results indicate that COMMD10 enhances ferroptosis and radiosensitivity in HCC by disrupting Cu-Fe homeostasis and inhibiting HIF1α/CP loop function (73). These findings indicate that gene sensitizing therapy can enhance the effectiveness of radiotherapy, decrease the dosage and adverse effects of radiotherapy, and improve the survival rate of HCC patients when combined with COMMD10 overexpression.

### 4.5 Immune microenvironment and related immunotherapy

In recent years, many studies have shown that tumor cell ferroptosis can reshape the tumor immune microenvironment. Immune cells can also promote tumor cell ferroptosis, thereby exerting anti-tumor effects (74).

CD8+ T cells are the main executors of adaptive immunity. In addition to maintaining the survival and expansion of CD4+ and CD8+ T cells, GPX4 acts as a regulator of ferroptosis to protect activated regulatory T cells from ferroptosis and plays a major role in the inhibition of anti-tumor immunity (75). CD8+ T cells are the major players in adaptive immunity. In addition to maintaining the survival and expansion of CD4+ and CD8+ T cells, GPX4 acts as a regulator of ferroptosis to protect activated regulatory T cells from ferroptosis and plays an important role in inhibiting anti-tumor immunity (76). IFN-γ, released by CD8+ T cells, alters the lipid composition of tumor cells via ACSL4 and induces an immunogenic ferroptosis of the tumor cells (77). A number of studies of targeted ferroptosis in combination with immune checkpoint blockade therapy have also shown that induction of ferroptosis in tumor cells in combination with anti-programmed death 1 (PD-1) antibody therapy has a strong anti-tumor effect (78). Treg cells are an immunosuppressive subset of CD4+ T cells that play an important role in maintaining self-tolerance and immune homeostasis. The abundance of Treg cells in the peripheral blood of patients with HCC was higher than that of patients without HCC (79). GPX4 is essential for Treg cells to maintain immune tolerance, and GPX4 deficiency causes excessive accumulation of lipid peroxides in Treg cells and triggers ferroptosis. In addition, GPX4 deficiency in Treg cells increased mitochondrial superoxide production and promoted IL-1β production. GPX4-specific knockdown of Treg cells suppressed tumor growth and prevented tumor immune escape without inducing significant autoimmunity (75).

Tumor-associated macrophages (TAMs) play an important role in recycling iron produced during red blood cell clearance. They are also an important component of host defense, protecting the host from the invasion of pathogens (80). M1 macrophages were more resistant to ferroptosis induced by GPX4 deletion. The M2 isoform is more susceptible to ferroptosis induced by GPX4 inhibition due to its lack of inducible nitric oxide synthase expression and nitric oxide production (81). APOC1 is overexpressed in TAMs of HCC tissues in comparison to normal tissues, and inhibition of APOC1 reverses the transition from M2 phenotype to M1 phenotype via the ferroptosis pathway, thereby enhancing the efficacy of anti-PD1 immunotherapy for HCC (82).

Pathologically activated neutrophil-myeloid-derived suppressor cells (PMN-MDSCs), downregulation of GPX4 promotes ferroptosis of PMN-MDSCs. Tumor PMN-MDSCs were more sensitive to ferroptosis and could mediate immunosuppression after ferroptosis compared to PMN-MDSCs isolated from bone marrow and spleen (83). Thus, ferroptosis represents a unique and targeted immunosuppressive mechanism of PMN-MDSCs in TEMs that can be pharmacologically modulated to limit the progression of tumors (84). Thus, ferroptosis is a unique and targeted PMN-MDSC immunosuppressive mechanism in TEMs that can be pharmacologically modulated to limit tumor progression.

Natural killer (NK) cell dysfunction leads to increased tumor incidence and growth rate. Dysfunctional NK cells in the TME were found to have increased expression of proteins associated with lipid peroxidation and oxidative damage, and cell morphology was similar to that of ferroptosis cells. Oxidative stress associated with lipid peroxidation may inhibit NK cell glucose metabolism, leading to NK cell dysfunction in the TME (85). The liver contains large numbers of hyporeactive NK cells, which are an important source of hepatic tolerance that can expose the body to enteric exogenous antigens on a daily basis without causing inflammation. We do not know whether NK cells themselves are subject to ferroptosis, but in a study of sepsis, researchers reported that many ferroptosis-related genes were dysregulated in models of liver failure and promoted liver failure by interacting with NK cells (86).

In conclusion, ferroptosis is expected to provide new ideas and targets to improve the efficacy of immunotherapy in HCC patients. Through a strategy of combining ferroptosis with immunotherapy, it brings new hope for the treatment of HCC. Nanotechnology has had remarkable success in cancer therapy. Studies have shown that in SRF@Hb-Ce6 nanoparticles, constructed with hemoglobin (Hb) linked to the photosensitiser chlorin e6 (Ce6) and loaded with sorafenib (SRF), Hb uses its own oxygen-bound iron to provide oxygen for photodynamic therapy (PDT) and iron for ferroptosis. PDT enhanced ferroptosis by inducing IFNγ secretion from immune cells (87). Carbon quantum dots (CQDs) are a class of zero-dimensional carbon nanomaterials with significant fluorescence capability. Yao et al. reported a CQD-based biocompatible nanoenzyme prepared from chlorogenic acid (ChA), a major bioactive natural product in coffee. ChACQDs induced ferroptosis through a GSH depletion-dependent ROS production mechanism, while attracting a large number of tumor-expanding immune cells, including T cells and NK cells, and activating systemic anti-tumor immune responses (88). Nanoparticles may therefore be an effective anti-tumor strategy. They induce ferroptosis and mobilize an immune response against HCC cells.

### 4.6 Combination therapy

Ferroptosis plays an important role in anti-tumor therapy, but it is often difficult to achieve the desired effect with a single treatment method. Therefore, the treatment of tumors is usually a combination of several treatment modalities, especially in advanced patients. Many studies have shown great potential for synergistic therapy by inducing ferroptosis in cancer cells in combination with other existing therapies. A. The combination of immunotherapy and targeted molecular therapy has shown promising therapeutic effects in HCC. For example, phosphoglycerate mutase 1 (PGAM1) has been identified as a novel immunometabolism target. Inhibition of PGAM1 exerts anti-tumor effects by promoting ferroptosis and CD8+ T-cell infiltration, and can significantly enhance the efficacy of anti-PD-1 immunotherapy in HCC through synergy with anti-PD-1 immunotherapy in HCC, providing preclinical evidence for the use of this combination therapy (89). Other studies have reported that combining ferroptosis induction with MDSC blockade sensitises primary and metastatic tumors in the liver to immune checkpoint blockade and can be used to treat HCC and liver metastases (90). B. In radiotherapy and molecular targeted therapy, SOCS2 acts as a bridge to transfer the bound ubiquitin to SLC7A11, promoting polyubiquitination and degradation of K48-associated SLC7A11, ultimately leading to ferroptosis and radiosensitivity of HCC. These results suggest that SOCS2-targeting drugs may be combined with radiotherapy to improve patient prognosis (71). C. Sorafenib plays a role by inducing ferroptosis in HCC cells, but sorafenib resistance has also been reported to be associated with ferroptosis in several studies. Upregulation of fatty acid synthase (FASN) antagonized SLC7A11-mediated ferroptosis, thereby promoting sorafenib resistance. The FASN inhibitor orlistat combined with sorafenib showed significant synergistic anti-tumor effects and reversed sorafenib resistance *in vitro* and *in vivo*. The combination of the two has excellent synergistic anti-tumor effects in sorafenib-resistant HCC cells (91). D. DHA loaded Fe-MnO2 nanosheets are used in ferroptosis and immunotherapy. DHA not only acts as an ferroptosis inducer, but also acts as an immunomodulator to inhibit Tregs to exert systemic anti-tumor effects. Fe-MnO2/DHA is a multimodal treatment for HCC driven by ferroptosis, apoptosis, and immune activation, which significantly improves the efficacy of synergistic cancer treatment (92).

## 5 Prospects

Research progress in ferroptosis has been a hot topic in recent years. Ferroptosis plays an important role in combating the occurrence and development of many diseases. This article mainly reviews the regulation of ferroptosis in HCC. As our insight into the regulation of ferroptosis in liver cancer continues to deepen, based on the study of ferroptosis, we have new ideas for the diagnosis and treatment of HCC, such as early diagnosis, new tumor classification, improvement in drug resistance, development of new drugs, and evaluation of disease prognosis. By reviewing the relevant references, we have confirmed that there are potential biomarkers of ferroptosis that can help in the early diagnosis and monitoring of HCC. Based on these biomarkers, researchers have conducted relevant in-depth research on the treatment of HCC. For example, the study by Deng et al. showed that coiledcoil containing domain containing 25 (CCDC25) may be a potential diagnostic and prognostic marker for HCC associated with immune infiltration and ferroptosis. In-depth studies have shown that downregulation of CCDC25 increases the sensitivity of HCC patients to a variety of drugs, including sorafenib (93). Yan et al. found that BAP1-mutant HCCS had a reduced ability to promote ferroptosis, suggesting that high BAP1 expression is associated with ferroptosis. They identified BAP1 as a potential diagnostic and prognostic marker for HCC and as a marker for ferroptosis and immune checkpoint blockade therapies (94). However, there are still a lot of questions that need to be answered, such as: A. How are the three main regulatory pathways of ferroptosis related to each other? B. The current ferroptosis-based drugs for HCC treatment are in the early stages of development, and many of them have not been used in the clinic. C. The direct targets of ferroptosis in HCC have not been fully defined. D. Induction of ferroptosis has a dual effect. When ferroptosis is induced in HCC cells, it also induces ferroptosis in other normal cells, which damages normal cells. How to better induce ferroptosis in HCC cells and avoid ferroptosis in other cells? Induction of ferroptosis has a dual effect. While it induces ferroptosis in HCC cells, it also induces ferroptosis in normal cells and damages normal cells. Therefore, how to effectively induce ferroptosis in HCC cells without affecting normal cells is an important consideration. We believe that molecular targeted therapy is the important part to solve this problem. Based on the differential expression of ferroptosis-related regulatory factors in HCC, the targeted drugs that regulate related factors can specifically induce ferroptosis in HCC cells without damaging or minimally damaging normal cells, and exert an effective anti-tumor effect. For example, Li et al. identified N6F11 as a ferroptosis inducer and found that N6F11 must bind to a specific domain of the ubiquitin E3 ligase (TRIM25) to trigger the ubiquitination degradation of GPX4. Since TRIM25 is mainly expressed in pancreatic cancer tumor cells and not in immune cells, this small molecule can selectively induce GPX4 protein degradation in pancreatic cancer tumor cells and induce T cell-mediated anti-tumor immunity without inducing ferroptosis in immune cells (95). Further research is needed to address these issues, particularly in the treatment of HCC, especially for patients who are not candidates for surgical resection. New drugs and solutions to drug resistance are also needed.

## Author contributions

SJ: Writing – original draft, Writing – review & editing. GZ: Writing – review & editing. YM: Supervision, Writing – review & editing. DW: Writing – review & editing. DX: Writing – review & editing. SZ: Writing – review & editing. XJ: Funding acquisition, Resources, Supervision, Writing – review & editing.
